# Influence of Daily Individual Meteorological Parameters on the Incidence of Acute Coronary Syndrome

**DOI:** 10.3390/ijerph111111616

**Published:** 2014-11-12

**Authors:** Mirjam Ravljen, Marjan Bilban, Lučka Kajfež-Bogataj, Tomaž Hovelja, Damjan Vavpotič

**Affiliations:** 1 Faculty of Health Sciences, University of Ljubljana, Zdravstvena Pot 5, SI-1000 Ljubljana, Slovenia; 2 Institute of Occupational Safety, Chengdujska Cesta 25, SI-1260 Ljubljana-Polje, Slovenia; E-Mail: marjan.bilban@zvd.si; 3 Biotechnical Faculty, University of Ljubljana, Jamnikarjeva 101, SI-1000 Ljubljana, Slovenia; E-Mail: lucka.kajfez.bogataj@bf.uni-lj.si; 4 Information Systems Laboratory, Faculty of Computer and Information Science, University of Ljubljana, Tržaška 25, SI-1000 Ljubljana, Slovenia; E-Mails: tomaz.hovelja@fri.uni-lj.si (T.H.); damjan.vavpotic@fri.uni-lj.si (D.V.)

**Keywords:** cardiovascular disease, meteorological factors, atmospheric pressure, humidity, temperature, myocardial infarction, weather, Europe

## Abstract

*Background*: A nationwide study was conducted to explore the short term association between daily individual meteorological parameters and the incidence of acute coronary syndrome (ACS) treated with coronary emergency catheter interventions in the Republic of Slovenia, a south-central European country. *Method*: We linked meteorological data with daily ACS incidence for the entire population of Slovenia, for the population over 65 years of age and for the population under 65 years of age. Data were collected daily for a period of 4 years from 1 January 2008 to 31 December 2011. In line with existing studies, we used a main effect generalized linear model with a log-link-function and a Poisson distribution of ACS. *Results and Conclusions*: Three of the studied meteorological factors (daily average temperature, atmospheric pressure and relative humidity) all have relevant and significant influences on ACS incidences for the entire population. However, the ACS incidence for the population over 65 is only affected by daily average temperature, while the ACS incidence for the population under 65 is affected by daily average pressure and humidity. In terms of ambient temperature, the overall findings of our study are in line with the findings of the majority of contemporary European studies, which also note a negative correlation. The results regarding atmospheric pressure and humidity are less in line, due to considerable variations in results. Additionally, the number of available European studies on atmospheric pressure and humidity is relatively low. The fourth studied variable—season—does not influence ACS incidence in a statistically significant way.

## 1. Introduction 

Since Rosahn in 1937 [1], a number of studies on almost all continents [2,3,4,5,6,7,8,9] have indicated that weather plays an important role in the onset of cardiovascular (CV) disease. The short-term effect on both CV morbidity and mortality has been evaluated through various analyses [10,11,12,13,14,15,16,17,18,19,20,21,22].

Several researchers have focused their attention on acute myocardial infarction (AMI), which is a common CV disease that requires emergency medical treatment. The impact of ambient temperature on AMI morbidity has received less attention in the past [23], while the influence of seasonal variations on the incidence of AMI has been well examined. However, these seasonal variations do not seem to be universal [24,25,26,27,28,29,30,31,32]. It is possible that there is geographical variation in the seasonal distribution of AMI [33]. Fewer studies are available on the influence of individual meteorological parameters on AMI [34,35] although a statistically significant effect of ambient temperature on AMI risk has been consistently reported [36,37,38]. Only a minority of them have been nationwide [39] as in our study.

Our study included patients who in the research period had received emergency treatment in catheterisation laboratories, with primary and rescue percutaneous coronary emergency catheter intervention. The population is suitable for investigation because the danger of the leading symptom of ACS, angina pectoris, is well known in Slovenia. We also have a good national survey of diagnoses leading to hospitalisation and the “door-to-balloon time”—a critical element in reperfusion therapy worldwide—is short. By focusing on the chosen group of patients, we wished to ensure a minimum time gap between the patient’s exposure to individual meteorological conditions, the onset of symptoms and the diagnostics and therapy applied.

The aim of the study was to examine the influence of daily individual meteorological parameters on the incidence of ACS in Slovenia and to compare the findings with other European studies. Additionally, we hope that the study results can improve hospital material and work schedule planning, as well as provide relevant weather alerts for people with heart-related conditions. 

## 2. Materials and Methods

Slovenia, a country with a population of two million people, is a central European country with an area of 20,000 km^2^, situated between Italy, Hungary, Austria and Croatia. The air temperature in Slovenia has a distinctive daily and yearly course and differences between seasons are pronounced.

**Figure 1:**
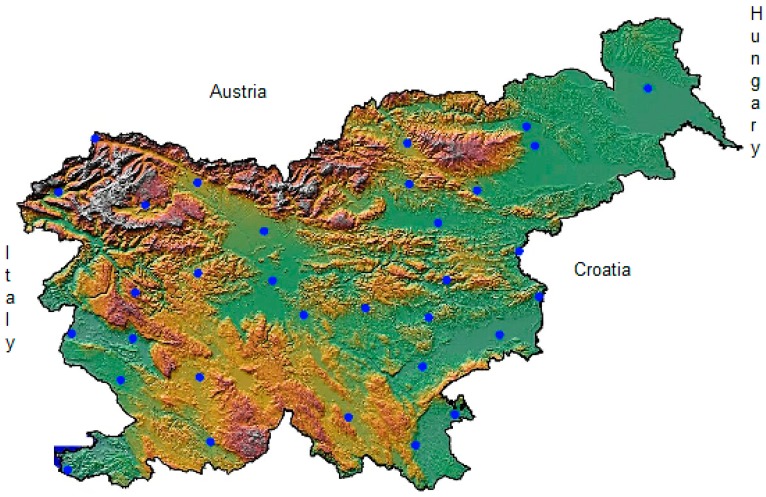
Meteorological stations in Slovenia [40].

Data on the incidence of ACS were obtained from all hospitals in Slovenia that have catheterization laboratories with 24-h medical teams for emergency treatment of patients. They are located in the three largest cities—Ljubljana, Maribor and Celje. Ambulance or helicopter emergency transport is used for transporting patients from remote locations directly to an interventional cardiac catheterization facility. Data were collected daily for a period of 4 years from 1 January 2008 to 31 December 2011. During the study, 6434 patients received emergency treatment in catheterisation laboratories, of which there were 4412 (69%) men and 2022 (31%) women; 2494 men (39%) and 612 (10%) women were younger than 65 years of age. Data on daily ambient temperatures, humidity and atmospheric pressure (meteorological data) were obtained from 32 meteorological stations of the Slovenian Environment Agency (Figure 1). 

Distance and altitude of a town were considered in choosing the appropriate station in terms of the patient’s address of permanent residence. The stations cover one or more municipalities in the three major geographical regions of Slovenia. To gain representative meteorological data for Slovenia, we summed the weighted station data. The weighted station data was computed by multiplying each station data by the percentage of the population that live in the municipalities covered by a specific station. The climatological data thus represents the climatological conditions for the population in Slovenia as accurately as possible.

Since not all of the 32 climatological stations used the same data format, special care had to be taken to ensure data consistency. Furthermore, in order to pair the climatological data with the incidence of ACS data properly, appropriate data handling and calculation techniques and tools had to be used. For merging and aggregating data R 3.0.0 (The R Foundation for Statistical Computing, Vienna, Austria) [41] with its basic packages was used while statistical analysis was done with SPSS 20.0 (IBM Corporation, Armonk, New York, NY, USA) [42]. 

To link data on the incidence of ACS and climatological data, we followed the well-established approach of a multivariate analysis based on a main effect generalized linear model, assuming a log-link function with a Poisson distribution (GLM-LL model) [7,37,43,44]. The Poisson distribution is a discrete distribution and is appropriate for counts of observations (number of acute coronary syndromes per day). 

## 3. Results 

In this section, the results of GLM-LL models are presented linking climatological data with the following: daily ACS incidence for the entire population, daily ACS incidence for the population over 65 years of age and daily ACS incidence for the population under 65 years of age. In order to investigate the seasonal effect of warm and cold periods on ACS incidence, we introduced a season variable into the model. The warm period (Season = 1) was defined as the period from April to September and the cold period (Season = 0) was defined as the period from October to March. 

Tests of over-dispersion confirmed the Poisson model assumptions for the three fitted models. In all three cases, the ratio of deviance to degrees of freedom, the ratio of the Pearson Chi-Square to degrees of freedom and the variance to mean ratio did not surpass 1.3. Additionally, statistical tests of the GLM-LL models showed statistically significant goodness of fit. In all three models, the *p*-value of the Omnibus test (Likelihood Ratio Chi-Square) was below 0.05. Such results indicate a relevant improvement of the fitted model over the intercept-only model. 

The results for daily ACS incidence for the entire population presented in Table 1 show that all the beta coefficients of the fitted model were statistically significantly different from 0, except the beta coefficient of the season variable. The per mille change of the beta coefficient is computed according to the formula Exp(B) – 1 = 0.993 – 1= −0.007. The same formula is used to compute the 95% confidence intervals (95% CI). The Exp function is used because the interpretation of beta coefficients of the GLM-LL model has to take into account their exponential nature. Thus, if the average daily temperature increases by 1 °C, the daily incidence of ACS decreases by approximately 7‰, with a 95% CI (−12‰, −2‰). If the daily average humidity increases by 1%, the daily incidence of ACS decreases by approximately 3‰, 95% CI (−6‰, −1‰). If the daily average pressure increases by 1 mbar, the daily incidence of ACS decreases by approximately 4‰, 95% CI (−7‰, −1‰). 

**Table 1:** Daily ACS incidence for the entire population.

Parameter	B	95% Confidence Interval		Hypothesis Test		Exp(B)	95% Confidence Interval for Exp(B)	
		Lower	Upper	Wald Chi-Square	Sig.		Lower	Upper
(Intercept)	5.9309	2.640	9.222	12.478	0.000	376.504	14.015	10,114.4
Average daily T (°C)	−0.0071	−0.012	−0.002	7.484	0.006	0.993	0.988	0.998
Average daily H (%)	−0.0031	−0.006	−0.001	5.854	0.016	0.997	0.994	0.999
Average daily P (mbar)	−0.0042	−0.007	−0.001	6.363	0.012	0.996	0.993	0.999
Season	0.0222	−0.062	0.106	0.268	0.605	1.022	0.940	1.112

Notes: Dependent variable: ACS incidence for the entire population; model: (intercept), average daily temperature (T) (°C), average humidity (H) (%), average pressure (P) (mbar), and season (warm period: April–September, cold period: October–March); Sig.: significance probability.

The results of daily ACS incidence for the population under 65 presented in Table 2 show that all the beta coefficients of the fitted model were statistically significantly different from 0, except the beta coefficient of average daily temperature and the beta coefficient of the season variable. If the daily average humidity increases by 1%, the daily incidence of ACS decreases by approximately 4‰, 95% CI (−7‰, −0.2‰). If the daily average pressure increases by 1 mbar, the daily incidence of ACS decreases by approximately 7‰, 95% CI (−11‰, −2‰).

**Table 2:** Daily ACS incidence for population under 65.

Parameter	B	95% Confidence Interval		Hypothesis Test		Exp(B)	95% Confidence Interval for Exp(B)	
		Lower	Upper	Wald Chi-Square	Sig.		Lower	Upper
(Intercept)	7.7627	3.050	12.476	10.422	0.001	2351.29	21.112	261,867.6
Average daily T (°C)	−0.0050	−0.012	0.002	1.770	0.183	0.995	0.988	1.002
Average daily H (%)	−0.0037	−0.007	0.000	4.198	0.040	0.996	0.993	0.9998
Average daily P (mbar)	−0.0068	−0.011	−0.002	8.011	0.005	0.993	0.989	0.998
Season	−0.0027	−0.123	0.118	0.002	0.965	0.997	0.884	1.125

Notes: Dependent variable: ACS incidence for population under 65 years of age; model: (intercept), average daily temperature (T) (°C), average humidity (H) (%), average pressure (P) (mbar), season (warm period: April–September, cold period: October–March); Sig.: significance probability.

The results of daily ACS incidence for the population over 65 presented in Table 3 show that the model’s intercepts, as well as the influences of average daily humidity, average daily pressure and season on daily ACS incidence for patients older than 65 years, were not statistically significant. 

The only statistically significant beta coefficient is thus average daily temperature. If the average daily temperature increases by 1 °C, the daily incidence of ACS decreases by approximately 9‰, 95% CI (−16‰, −2‰). Based on the three GLM-LL models presented, it appears that daily average temperature has an impact on ACS incidence for the entire population as well as the population over 65. Humidity and pressure, on the other hand, impact on the ACS incidence of the entire population and the population of people under 65. 

**Table 3:** Daily ACS incidence for population over than 65 years.

Parameter	B	95% Confidence Interval		Hypothesis Test		Exp(B)	95% Confidence Interval for Exp(B)	
		Lower	Upper	Wald Chi-Square	Sig.		Lower	Upper
(Intercept)	2.8726	−1.724	7.469	1.501	0.221	17.682	0.178	1752.3
Average daily T (°C)	−0.0091	−0.016	−0.002	6.311	0.012	0.991	0.984	0.998
Average daily H (%)	−0.0024	−0.006	0.001	1.911	0.167	0.998	0.994	1.001
Average daily P (mbar)	−0.0018	−0.006	0.003	0.607	0.436	0.998	0.994	1.003
Season	0.0455	−0.071	0.162	0.582	0.445	1.047	0.931	1.176

Notes: Dependent variable: ACS incidence for population over 65; model: (intercept), average daily temperature (T) (°C), average humidity (H) (%), average pressure (P) (mbar), season (warm period: April–September, cold period: October–March); Sig.: significance probability.

## 4. Discussion

The discussion is organised into four main parts, each presenting one of the key variables of our model. Our findings regarding each variable are compared to contemporary studies. Most studies focus on temperature while only a few consider other meteorological variables.

Contemporary European studies conducted in France [36], Germany [45], Italy [43], Hungary [39] , England [46] and Denmark [23] report a negative correlation between average daily temperature and the incidence of AMI, as in our study. In Portugal Vasconcelos *et al.* [47] also reported a negative correlation, although they used various indices rather than individual meteorological variables. On the other hand, Wijnbergen *et al.* from The Netherlands [48] and Goerre *et al.* from Switzerland [35] report that incidences of AMI do not statistically significantly differ between colder days and warmer days. Similarly, a study of hospitals in a Turkish city and patients over the age of 65 did not confirm any connection between incidence and low temperatures [49]. Many recent studies have also established differences in terms of age [4,36,45,46,50] regarding the incidence of AMI at low temperatures. Our study also confirmed the effect of cold temperatures being more pronounced among the older population.

The few European studies that included pressure in their analysis found contradicting results. Some did not find any relation between pressure and the incidence of AMI [43,48,51,52] or the relationship was not statistically significant [52]. Others describe either a positive correlation [35,36] or, as in our study, a negative correlation [36,47].

There are also not many studies evaluating the influence of humidity on the incidence of AMI. Ruhenstroth-Bauer *et al.* [3] from Germany established a similarly negative correlation as our study. On the other hand, Panagiotakos *et al.* [50] from Greece, Abrignani *et al.* [43] from Italy and Ezekowitz *et al.* [53] in an international study report positive correlations. Barnett *et al.* [54] and Wijnbergen *et al.* [48] reported that the effect of humidity was not statistically significant. The results in relation to humidity are less comparable because some authors use maximal ambient humidity and others use relative humidity in their research. 

Many studies studying the seasonal effects on AMI incidences found statistically significant influences [24,27,28,30], while some studies found only partial influences [25,26] and others do not report statistically significant seasonal effects [4,29,31,55]. Our results also show that the season does not statistically significantly influence ACS incidences. Our findings are aligned with the findings of Marchant *et al.* [56], who report that AMI was more common on colder days, independent of the season. Moreover, Slovenia doesn’t experience extreme cold and warm seasons, which agrees with Ku *et al.* [55], who also found no variations in a region lacking temperature extremes. 

## 5. Conclusions

The overall findings of our study correspond to the findings of other European studies in terms of temperature. In light of the proven major effect of lower temperatures on the risk of the onset of ACS, even more attention and preventive measures should be directed into this field. While there are some major differences in relation to humidity and pressure among published European studies, there have also been far fewer studies that analyse this effect. It has also been established that the incidence rate differs among different countries. This probably cannot be explained only by conventional risk factors and temperature, since there are also other factors, including other meteorological variables, which may play an important part. Further research should also include an analysis of the time that individuals actually spend outdoors. 

The results of our study suggest that more attention should be focused on the negative effect of low temperatures, low pressure and low humidity. Warning systems considering all these variables should be developed similarly to existing hot weather warning systems [57]. Our future work will focus on the potential of developing a mobile application with a corresponding back-end system providing relevant alerts for people with heart-related conditions. Another avenue of further work is the improvement of hospital material and work schedule planning through the integration of meteorological data.
